# The appropriate dosing of erenumab for migraine prevention after multiple preventive treatment failures: a critical appraisal

**DOI:** 10.1186/s10194-019-1054-4

**Published:** 2019-10-30

**Authors:** Raffaele Ornello, Cindy Tiseo, Ilaria Frattale, Giulia Perrotta, Carmine Marini, Francesca Pistoia, Simona Sacco

**Affiliations:** 1 Neuroscience Section, Department of Applied Clinical Sciences and Biotechnology, University of L’Aquila, Via Vetoio 1, 67100 L’Aquila, Italy; 2 Department of Life, Health and Environmental Sciences, University of L’Aquila, L’Aquila, Italy

**Keywords:** Migraine, Calcitonin gene-related peptide, Migraine prevention, Monoclonal antibodies, Erenumab

## Abstract

**Background:**

Erenumab, a fully human monoclonal antibody directed against the calcitonin gene-related peptide receptor, was approved for the prevention of episodic (EM) or chronic migraine (CM) at the monthly dose of 70 mg or 140 mg. We reviewed the available literature to understand if patients with prior preventive treatment failures benefit more from the 140 mg dose than the 70 mg.

**Main body:**

We searched papers indexed in PubMed and conference abstracts published in the last 2 years which assessed the safety and efficacy of erenumab in patients with prior preventive treatment failures. We reviewed the results of 3 randomized controlled trials and their subgroup analyses and open-label extensions. The 140 mg monthly dose of erenumab had a numerical advantage over the 70 mg monthly dose in patients with prior preventive treatment failures, both in EM and CM (with or without medication overuse) during the double blind phases of the trials and their open-label extensions. The numerical difference between the two doses increased with the increase in the number of prior preventive treatment failures.

**Conclusions:**

The available data suggest that erenumab 140 mg monthly might be preferred over the 70 mg monthly dose in patients with EM or CM and prior preventive treatment failures. Further data are needed to assess the long-term efficacy in clinical practice of the two doses of erenumab, while their safety profile is comparable.

## Background

Monoclonal antibodies acting on the calcitonin gene-related peptide (CGRP) or its receptor are the first specific treatment for the prevention of episodic (EM) or chronic migraine (CM). The use of those drugs has raised great expectations [1], and their efficacy and excellent safety make them suitable for most migraineurs. However, due to their high cost, patient selection is an important issue [2, 3]. It has been suggested that their use may be firstly considered for those with disabling attacks or for those in the pre-chronic stage of the disease to prevent CM [4, 5]. The *American Headache Society* [6] and the *European Headache Federation* [7] currently recommend monoclonal antibodies acting on the CGRP or its receptor in patients who failed at least two of the available preventive treatments.

Erenumab, a fully human monoclonal antibody directed against the CGRP receptor, was approved for the prevention of EM or CM at the monthly dose of 70 or 140 mg; the 70 mg monthly dose is recommended in most patients with migraine, while the 140 mg dose provides an additional benefit to some patients [8]. We performed a critical appraisal of the available literature to understand if patients who had failed prior preventive treatments may benefit more from the 140 mg erenumab dose than the 70 mg.

## Methods

We searched papers indexed in PubMed over the last 2 years which contained the terms ‘migraine’ and ‘erenumab’ in their title or abstract. We also manually searched conference abstracts published over the same time span. Papers and abstracts were eligible for this review if they reported about the effect of erenumab in patients with and without prior preventive treatment failures.

## Review of the available trials

Detailed information on patients with prior preventive treatment failures was available from 3 randomized controlled trials (RCTs) – the NCT02066415, the Study to Evaluate the Efficacy and Safety of Erenumab (AMG 334) in Migraine Prevention (STRIVE), and the 12-week Double-blind, Randomized, Multicenter Study Comparing the Efficacy and Safety of Once Monthly Subcutaneous AMG 334 Against Placebo in Adult Episodic Migraine Patients Who Have Failed Prophylactic Migraine Treatments (LIBERTY) (Table 1) [9, 14, 17] - and their subgroup analyses or open-label extensions (OLEs) [10–13, 15, 16, 18, 19].

**Table 1:** Characteristics of the randomized controlled trials assessing the effect of prior preventive treatment failures on the treatment with erenumab for migraine prevention

	NCT02066415 [9–13]	STRIVE [14–16]	LIBERTY [17, 18]
Phase	II	III	IIIb
Migraine type	CM	EM	EM
Dose (mg)	70/140	70/140	140
OLE duration (weeks)	52	52	24
Duration of blind phase (weeks)	12	24	12
Prior preventive treatment failures^a^ accepted	≤3	≤2	2–4
No. of patients			
Placebo	286	319	125
70 mg	191	317	–
140 mg	190	319	121
Patients with prior preventive treatment failure(s) (%)			
Placebo	70	40	100
70 mg	67	40	–
140 mg	66	36	100

*MMD* Monthly Migraine Days, *OLE* Open-Label Extension, *EM* Episodic Migraine, *CM* Chronic Migraine

^a^due to lack of response; partial response or suspension due to tolerability were accepted

The STRIVE trial considered 7 categories of preventive treatments, namely: 1) divalproex sodium, sodium valproate; 2) topiramate; 3) beta blockers; 4) tricyclic antidepressants; 5) serotonin-norepinephrine reuptake inhibitors; 6) flunarizine, verapamil; and 7) lisinopril, candesartan [14]; the NCT02066415 trial considered the same categories plus botulinum toxin [9], while the LIBERTY trial included in migraine prophylaxis treatments propranolol/metoprolol, topiramate, flunarizine, valproate/divalproex, amitriptyline, venlafaxine, lisinopril, candesartan, and locally approved products (e.g. oxeterone or pizotifen) [17].

### Episodic migraine

In the STRIVE trial [14], information on patients with prior preventive treatment failures came from subgroup analyses [15]. In that study, patients who failed more than ≥2 preventive drug categories were excluded, while the LIBERTY study included only patients with 2 to 4 prior treatment failures [17].

**Fig. 1:**
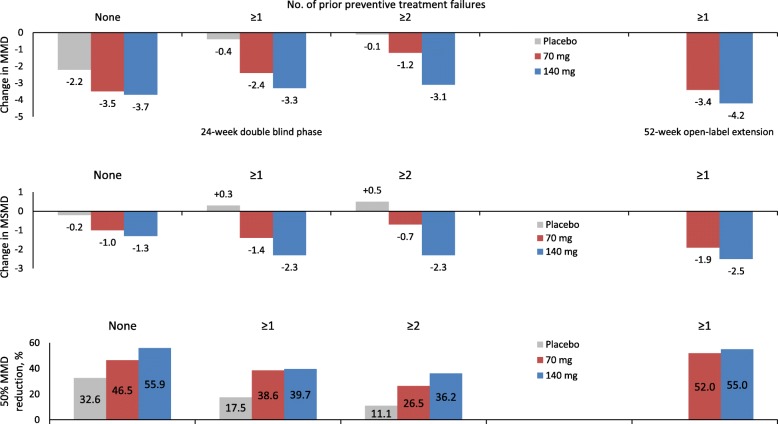
Patients with episodic migraine: effect of erenumab on monthly migraine days, monthly migraine-specific medication days, and 50% reduction in monthly migraine days from baseline according to prior preventive treatment failures in the STRIVE trial [15] and its open-label extension [16]

In the STRIVE trial, both the 70 and the 140 mg doses of erenumab performed significantly better than placebo in patients in whom ≥1 and ≥ 2 preventive treatment categories had failed (Fig. 1). The advantages of erenumab over placebo increased with the increase in the number of preventive treatment failures due to the decrease in the placebo effect. Notably, the effect of the 140 mg dose remained stable independent of the number of prior preventive treatment failures while the effect of the 70 mg decreased with the increasing number of failures (Fig. 1) [15]. The preliminary data of the OLE of the same trial (Fig. 1) showed that, in patients with ≥1 prior preventive treatment failure(s), the numerical advantage of the 140 mg over the 70 mg monthly dose in terms of monthly migraine days and acute medication days was sustained until week 52 [16].

In the LIBERTY trial, the 140 mg monthly dose of erenumab significantly increased the proportion of EM patients experiencing a ≥ 50% reduction in monthly migraine days compared with placebo (30% vs 17%) [17], and the preliminary OLE data showed that the effect was sustained over 24 weeks [18].

### Chronic migraine

In the 12-week NCT02066415 trial [9], patients were treated with erenumab 70 or 140 mg; information on patients with prior preventive treatment failures came from prespecified subgroup analyses [10]; in this study patients who failed more than 2 preventive drug categories were excluded.

**Fig. 2:**
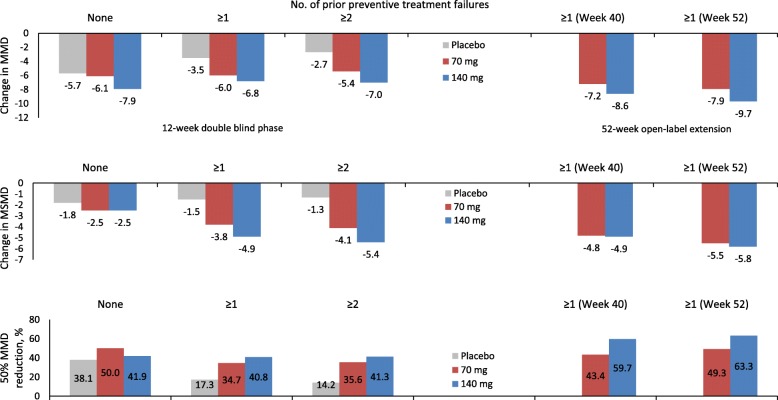
Patients with chronic migraine: effect of erenumab on monthly migraine days, monthly migraine-specific medication days, and 50% reduction in monthly migraine days from baseline according to prior preventive treatment failures in the NCT02066415 trial [10] and its open-label extension [11]

Even in CM patients, both doses of erenumab were significantly better than placebo in the subgroup of subjects with ≥1 and ≥ 2 preventive treatment category failures. Again, the advantage of erenumab over placebo increased with the increase in the number of preventive treatment failures due to decreasing placebo effect (Fig. 2) [10]. In the case of CM, even in the absence of direct comparisons, the 140 mg dose appeared numerically more advantageous than the 70 mg dose but there was not decreasing efficacy of the 70 mg dose with increasing number of treatment failures. Additionally, the CM-to-EM conversion rates over 12 weeks were similar with erenumab 70 mg or 140 mg compared with placebo (52.4% and 52.0% vs 28.9%) in CM patients with ≥1 prior preventive treatment failure(s) [19].

A long-term analysis of the trial OLE showed that patients with ≥1 prior preventive treatment failure(s) benefited from a sustained numerical advantage of the 140 mg monthly dose over the 70 mg one at Week 52 (Fig. 2) [11].

**Fig. 3:**
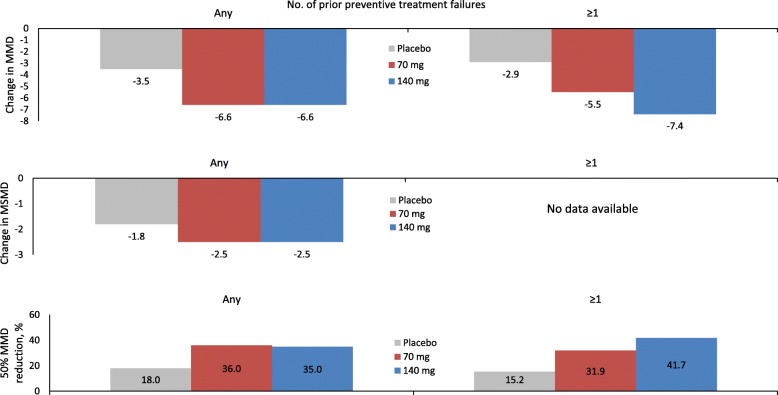
Patients with chronic migraine and medication overuse: effect of erenumab on monthly migraine days, monthly migraine-specific medication days, and 50% reduction in monthly migraine days from baseline according to prior preventive treatment failures in the NCT02066415 trial [12, 13]

A subgroup analysis of the NCT02066415 trial showed a comparable efficacy of the 70 mg and 140 mg monthly doses in patients with CM and medication overuse [12]; however, in those with ≥1 prior preventive treatment failure(s) the 140 mg monthly dose conferred an advantage over the 70 mg dose [13] (Fig. 3).

## Discussion

There are no data directly comparing the efficacy of the 70 and 140 mg erenumab dosing nor randomized dose-escalation studies. However, raw numbers suggest a slight clinical advantage of the 140 mg monthly dose of erenumab over the 70 mg monthly dose, which is more evident with increasing prior preventive treatment failures and in subjects with EM [10, 15]. This may suggest that treatment, in patients with prior preventive failures should be started or soon increased to the 140 mg dose. This treatment strategy will be feasible without additional costs or patient discomfort, since erenumab will be commercially available also in a single 140 mg injection [8]. Besides, no dose-related side effects were reported from the RCTs and their OLEs [9, 10, 14, 15, 20]. It is important to note that the LIBERTY trial included patients with EM with inadequate response, insufficient dosage or adverse events to 2–4 prior preventive treatments, and that this trial examined the 140 mg dose [17].

In the clinical practice setting erenumab is offered to patients not responding to multiple prior preventive treatments or unable to tolerate them because of lack of efficacy. Indeed, the *European Headache Federation* guidelines [7] and the position statement of the *American Headache Society* [6] recommend the use of monoclonal antibodies acting on the CGRP or its receptor in patients who failed ≥2 available preventive treatments because of lack of efficacy and/or poor tolerability, in order to limit the use of the costly novel migraine preventive treatments to the patients who need them most. Therefore, the profile of the patients who will be treated in the daily clinical practice will mainly match that of the LIBERTY study, which used the 140 mg dose only. Notably, the numerical advantage of the 140 mg dose over the 70 mg among patients with CM and prior preventive treatment failure(s) was independent of the presence of medication overuse [12, 13].

The selection of the right dose of erenumab for the right patient is still an open issue. It is important to act rapidly and effectively on the symptoms and on the progression of migraine and its associated disability. In RCTs, a relevant proportion of treatment failures was attributable to an insufficient dosage or to adverse events, rather than to a lack of response; besides, all RCTs except LIBERTY included a proportion of patients taking erenumab as their first preventive treatment (Table 1). We have no information on response to treatment for patients who failed previous preventatives versus those who did not tolerate them. Those who failed may represent a more difficult to treat subgroup as compared to those who did not tolerate treatment. Further data are also needed to assess whether the response to different erenumab doses of CM is different from that of EM. More data are also needed to assess whether the benefit of the 140 mg dose over the 70 mg one in patients with multiple prior preventive treatment failures is sustained over time; however, the available preliminary data regarding patients with ≥1 prior treatment failure(s) suggest a prolonged benefit [11, 16]. Notably, there are no data to indicate dose switch from 70 mg to 140 mg monthly, since in the available RCTs patients started and continued treatment with one of the two doses. Only some patients enrolled in the NCT02066415 trial switched doses 4 to 28 weeks after the double blind phase [11], and no analyses are available for that subgroup.

## Conclusions

According to the available data, the 140 mg monthly dose of erenumab should be preferred over the 70 mg dose in patients with migraine and prior preventive treatment failures, which is the target population according to available guidelines for prevention of migraine with monoclonal antibodies acting on the CGRP or its receptor. Further open-label and real-world studies will address the long-term benefit of starting – or switching to – the treatment with high-dose erenumab.

## Data Availability

All data operated or analyzed during this study are included in this published article.

## Abbreviations

CGRP: Calcitonin gene-related peptide
CM: Chronic migraine
EM: Episodic migraine
LIBERTY: A 12-week Double-blind, Randomized, Multicenter Study Comparing the Efficacy and Safety of Once Monthly Subcutaneous AMG 334 Against Placebo in Adult Episodic Migraine Patients Who Have Failed Prophylactic Migraine Treatments
MMDs: Monthly headache days
OLE: Open-label extension
RCT: Randomized controlled trial
STRIVE: Study to Evaluate the Efficacy and Safety of Erenumab (AMG 334) in Migraine Prevention

## References

[1] Martelletti P, Edvinsson L, Ashina M (2019). Shaping the future of migraine targeting calcitonin-gene-related-peptide with the disease-modifying migraine drugs (DMMDs). J Headache Pain.

[2] Tiseo C, Ornello R, Pistoia F, Sacco S (2019). How to integrate monoclonal antibodies targeting the calcitonin gene-related peptide or its receptor in daily clinical practice. J Headache Pain.

[3] Negro Andrea, Martelletti Paolo (2019). Patient selection for migraine preventive treatment with anti-CGRP(r) monoclonal antibodies. Expert Review of Neurotherapeutics.

[4] Martelletti P (2019). Erenumab is effective in reducing migraine frequency and improving physical functioning. BMJ Evid Based Med.

[5] Martelletti P (2017). The application of CGRP(r) monoclonal antibodies in migraine spectrum: needs and priorities. BioDrugs.

[6] American Headache Sociey (2019). The American headache society position statement on integrating new migraine treatments into clinical practice. Headache.

[7] Sacco S, Bendtsen L, Ashina M, Reuter U, Terwindt G, Mitsikostas DD, Martelletti P (2019). European headache federation guidelines on the use of monoclonal antibodies acting on the calcitonin gene related peptide or its receptor for migraine prevention. J Headache Pain.

[8] Aimovig, INN-Erenumab. European Medicines Agency (2019). https://www.ema.europa.eu/documents/overview/aimovig-epar-summary-public_en.pdf. Accessed 24 June 2019.

[9] Tepper S, Ashina M, Reuter U, Brandes JL, Doležil D, Silberstein S, Winner P, Leonardi D, Mikol D, Lenz R (2017). Safety and efficacy of erenumab for preventive treatment of chronic migraine: a randomised, double-blind, placebo-controlled phase 2 trial. Lancet Neurol.

[10] Ashina M, Tepper S, Brandes JL, Reuter U, Boudreau G, Dolezil D, Cheng S, Zhang F, Lenz R, Klatt J, Mikol DD (2018). Efficacy and safety of erenumab (AMG334) in chronic migraine patients with prior preventive treatment failure: a subgroup analysis of a randomized, double-blind, placebo-controlled study. Cephalalgia.

[11] Ashina M, Tepper SJ, Brandes J, Reuter U, Boudreau G, Doležil D, Klatt J, Zhang F, Cheng S, Mikol DD (2018) Long-term efficacy of erenumab in subjects with chronic migraine who failed prior prophylactic treatment. AAN Annual Meeting, 2018 Poster #PF106LB

[12] Tepper SJ, Diener HC, Ashina M, Brandes JL, Friedman DI, Reuter U, Cheng S, Nilsen J, Leonardi DK, Lenz RA, Mikol DD (2019). Erenumab in chronic migraine with medication overuse: subgroup analysis of a randomized trial. Neurology.

[13] Dodick D, Tepper SJ, Diener HC, Tassorelli C, Luas S, Evers S, Zhang F, Chou D, Tenenbaum N, Klatt J, Mikol D, Paiva da Silva Lima G (2019) Efficacy of erenumab in chronic migraine patients with medication overuse and prior preventive treatment failure. AAN 2019 Annual Meeting [Abstract]

[14] Goadsby PJ, Reuter U, Hallström Y, Broessner G, Bonner JH, Zhang F, Sapra S, Picard H, Mikol DD, Lenz RA (2017). A controlled trial of erenumab for episodic migraine. N Engl J Med.

[15] Goadsby PJ, Paemeleire K, Broessner G, Brandes J, Klatt J, Zhang F, Picard H, Lenz R, Mikol DD (2019). Efficacy and safety of erenumab (AMG334) in episodic migraine patients with prior preventive treatment failure: a subgroup analysis of a randomized, double-blind, placebo-controlled study. Cephalalgia.

[16] Reuter U, Schwedt TJ, Kudrow D, Paemeleire K, Zhang F, Klatt J, Picard H, Chou DE, Mikol DD (2019) Long-term efficacy of erenumab in patients with episodic migraine who have failed prior preventive migraine therapies. AAN 2019 Annual Meeting, Poster #10-020

[17] Reuter U, Goadsby PJ, Lanteri-Minet M, Wen S, Hours-Zesiger P, Ferrari MD, Klatt J (2018). Efficacy and tolerability of erenumab in patients with episodic migraine in whom two-to-four previous preventive treatments were unsuccessful: a randomised, double-blind, placebo-controlled, phase 3b study. Lancet.

[18] Reuter U, Goadsby P, Lanteri-Minet M, Hours-Zesinger P, Fernandes C, Ferrari M, Klatt J (2019) Assessment of the efficacy of erenumab during the open-label treatment (13–24 weeks) of subjects with episodic migraine who failed 2–4 prior preventive treatments: results of the LIBERTY study. AAN 2019 Annual Meeting [Abstract]

[19] Lipton RB, Tepper SJ, Silberstein S, Kudrow D, Ashina M, Reuter U, Dodick D, Zhang F, Rippon GA, Mikol DD (2019) Conversion from chronic to episodic migraine with erenumab, a specific inhibitor of the calcitonin gene-related peptide receptor. AAN 2019 Annual Meeting, Poster #PS25

[20] Ashina Messoud, Goadsby Peter J, Reuter Uwe, Silberstein Stephen, Dodick David, Rippon Gregory A, Klatt Jan, Xue Fei, Chia Victoria, Zhang Feng, Cheng Sunfa, Mikol Daniel D (2019). Long-term safety and tolerability of erenumab: Three-plus year results from a five-year open-label extension study in episodic migraine. Cephalalgia.

